# DNA-PKcs modulates progenitor cell proliferation and fibroblast senescence in idiopathic pulmonary fibrosis

**DOI:** 10.1186/s12890-019-0922-7

**Published:** 2019-08-29

**Authors:** David M. Habiel, Miriam S. Hohmann, Milena S. Espindola, Ana Lucia Coelho, Isabelle Jones, Heather Jones, Richard Carnibella, Isaac Pinar, Freda Werdiger, Cory M. Hogaboam

**Affiliations:** 1 Department of Medicine, Cedars-Sinai Medical Center, Women’s Guild Lung Institute, 127 S San Vicente Blvd., AHSP A9315, Los Angeles, CA 90048 USA; 2 Laboratory of Dynamic Imaging, Mechanical and Aerospace Engineering, Monash University, Clayton, VIC 3800 Australia

**Keywords:** DNA-PKcs, Mesenchymal progenitor cells, Senescence, IPF

## Abstract

**Background:**

Recent studies have highlighted the contribution of senescent mesenchymal and epithelial cells in Idiopathic Pulmonary Fibrosis (IPF), but little is known regarding the molecular mechanisms that regulate the accumulation of senescent cells in this disease. Therefore, we addressed the hypothesis that the loss of DNA repair mechanisms mediated by DNA protein kinase catalytic subunit (DNA-PKcs) in IPF, promoted the accumulation of mesenchymal progenitors and progeny, and the expression of senescent markers by these cell types.

**Methods:**

Surgical lung biopsy samples and lung fibroblasts were obtained from patients exhibiting slowly, rapidly or unknown progressing IPF and lung samples lacking any evidence of fibrotic disease (i.e. normal; NL). The expression of DNA-Pkcs in lung tissue was assessed by quantitative immunohistochemical analysis. Chronic inhibition of DNA-PKcs kinase activity was mimicked using a highly specific small molecule inhibitor, Nu7441. Proteins involved in DNA repair (stage-specific embryonic antigen (SSEA)-4^+^ cells) were determined by quantitative Ingenuity Pathway Analysis of transcriptomic datasets (GSE103488). Lastly, the loss of DNA-PKc was modeled in a humanized model of pulmonary fibrosis in NSG SCID mice genetically deficient in *PRKDC* (the transcript for DNA-PKcs) and treated with Nu7441.

**Results:**

DNA-PKcs expression was significantly reduced in IPF lung tissues. Chronic inhibition of DNA-PKcs by Nu7441 promoted the proliferation of SSEA4^+^ mesenchymal progenitor cells and a significant increase in the expression of senescence-associated markers in cultured lung fibroblasts. Importantly, mesenchymal progenitor cells and their fibroblast progeny derived from IPF patients showed a loss of transcripts encoding for DNA damage response and DNA repair components. Further, there was a significant reduction in transcripts encoding for *PRKDC* (the transcript for DNA-PKcs) in SSEA4^+^ mesenchymal progenitor cells from IPF patients compared with normal lung donors. In SCID mice lacking DNA-PKcs activity receiving IPF lung explant cells, treatment with Nu7441 promoted the expansion of progenitor cells, which was observed as a mass of SSEA4^+^ CgA^+^ expressing cells.

**Conclusions:**

Together, our results show that the loss of DNA-PKcs promotes the expansion of SSEA4^+^ mesenchymal progenitors, and the senescence of their mesenchymal progeny.

**Electronic supplementary material:**

The online version of this article (10.1186/s12890-019-0922-7) contains supplementary material, which is available to authorized users.

## Background

Interstitial lung diseases (ILD) are a group of diseases characterized by interstitial fibrosing of the lungs leading to the loss of alveolar function and normal gas exchange [1]. Idiopathic pulmonary fibrosis (IPF) is the most common form of ILD, with a median survival of 2–3 years after diagnosis [2] and limited therapeutic options. Currently, IPF patients are treated with OFEV or Esbriet, both of which slow down the progression of this disease, but many patients ultimately require lung transplantation despite treatment with these drugs. Most IPF patients that succumb to this disease die due to respiratory failure, however approximately 23% of these patients succumb due to non-respiratory causes, including ischemic heart failure, bronchogenic carcinoma, infection and pulmonary embolism [2]. Several studies have examined mechanisms leading to disrepair in IPF lung tissues and prevailing mechanisms suggest that epithelial dysfunction and/or progenitor cell abnormalities leads to chronic and aberrant activation of mesenchymal cells that are paramount to disrepair in lung fibrosis [3–5]. In line with this, a subpopulation of cells expressing the stage-specific embryonic antigen (SSEA)-4 with the properties of mesenchymal progenitors were identified in the lungs of IPF patients and shown to be “disease-mediating” [6]. Unlike their normal counterparts, IPF mesenchymal progenitor cells produce daughter cells that manifest the full spectrum of IPF hallmarks, including the ability to form fibrotic lesions in vivo [6]. Interestingly, in cancer, progenitor cells also have pathological roles by dividing asymmetrically and producing malignant daughter cells [7]. Although mesenchymal progenitor cells mediate repair and regeneration in response to injury, in IPF these cells may actually contribute to disrepair and disease progression.

Several studies have identified the relationship between DNA damage, DNA repair, and pulmonary fibrosis [8]. In fact, we have recently reported that loss of clusterin protein promoted disrepair and senescence in fibrotic lungs via the loss of DNA damage response and repair pathways [9]. DNA-dependent protein kinase catalytic subunit (DNA-PKcs), a member of the PI3K related family of enzymes, is a master regulator of the DNA-damage response [10]. DNA-PK is a complex of three proteins, consisting of Ku70/Ku80 and the catalytic holoenzyme DNA-PKcs, and is activated upon binding of Ku70/Ku80 and DNA. Activation induces its autophosphorylation as well as the phosphorylation of several other proteins that culminates in cell cycle checkpoint activation and DNA repair [11, 12]. DNA-PKcs is also integral to NHEJ DNA repair, VD-J recombination, anti-viral responses against DNA viruses, innate DNA sensing and in telomere maintenance [11–13]. Given the importance of this protein in DNA double strand break repair and cell cycle checkpoint, many studies have assessed its role in tumorigenesis. It has been shown that the kinase activity of DNA-PKcs can act as a negative regulator of p53-mediated CDKN1A transcription in stressed cells, and subsequently promoting apoptosis of the affected cells [14]. However, DNA-PKcs mediated NHEJ repair was observed to promote radiation resistance of various tumor cells [15]. Considering our previous data showing the relevance of DNA damage and loss of DNA damage repair pathways in disrepair and senescence in fibrotic lungs [9] and the divergent activities of DNA-PKcs in physiological and pathological settings, the objective of this study was to investigate the role of DNA-PKcs in IPF and lung fibrosis.

In this study we expand upon our previous observations and demonstrate the loss of DNA-PK in IPF lung tissues compared to nonfibrotic lungs. The expression of DNA-PKcs was rarely detected in the nuclei of IPF mesenchymal cells and it did not colocalize with hypomethylated CpG DNA (CpG), nor did it regulate CpG-mediated myofibroblast differentiation in normal or IPF lung fibroblasts. Chronic inhibition of DNA-PKcs kinase activity using a highly specific small molecule inhibitor, Nu7441 [16] promoted the expansion of SSEA4^+^ mesenchymal progenitor cells and a significant increase in the expression of myofibroblast, senescence-associated and inflammatory markers in cultured lung fibroblasts. Importantly, mesenchymal progenitor cells and their fibroblast progeny derived from IPF patients showed a loss of transcripts encoding for DNA damage response and DNA repair components. Further, there was a significant reduction in transcripts encoding for *PRKDC* (the transcript for DNA-PKcs) in SSEA4^+^ mesenchymal progenitor cells from IPF patients compared with normal lung donors. Transcriptomic, flow cytometric and immunofluorescence analysis suggested that SSEA4^+^ mesenchymal progenitor cells expressed the neuroendocrine marker, CgA. In a humanized SCID mouse model of IPF [17], treatment with Nu7441 promoted the expansion of mesenchymal progenitor cells (observed as a mass of SSEA4^+^ CgA^+^ expressing cells). Collectively, our results suggest that DNA-Pkcs expression is decreased in the lungs of IPF patients and its loss promotes the expansion of mesenchymal progenitor cells and increased expression of senescent markers in mesenchymal cells.

## Methods

### Patient samples and primary lung fibroblasts

Normal and IPF surgical lung biopsy samples and lung fibroblasts were obtained as previously described [17, 18]. Both normal and IPF lung fibroblasts were obtained by mechanically dissociating lung biopsy specimens or explants into sterile tissue culture plates and pipetting at least 10 times in complete medium [Dulbecco’s modified Eagle’s medium (DMEM; Lonza, Basel, Switzerland) plus 15% fetal bovine serum (Atlas Biologicals, Inc., Fort Collins, CO), 100 IU of penicillin, 100 μg/mL of streptomycin (Mediatech, Manassas, VA), 292 μg/mL of l-glutamine (Mediatech), and 100 μg/mL of Primocin (InvivoGen, San Diego, CA)]. Cells were maintained at 37 °C and 10% CO_2_ and medium was changed twice a week by carefully aspirating the liquid, retaining the minced tissues in the flask, and replacing with fresh medium. This process was repeated until stromal colonies were apparent (1 to 2 weeks), after which the colonies were trypsinized and passaged 4 to 5 times and fibroblast purity was confirmed by flow cytometry and/or real-time quantitative PCR analysis for CD45, EpCAM, and CD31.

Normal lung fibroblasts were derived from nonfibrotic lung samples lacking any evidence of disease. Patients were diagnosed with IPF using a multidisciplinary, clinical, radiological, and pathological mechanism [19]. The physiological criteria used to validate disease progression during the first year of follow-up included a forced vital capacity (FVC) decrease of greater than or equal to 10% and a diffusing capacity for carbon monoxide (DLCO) decrease of greater than or equal to 15% based on baseline physiological abnormality. Baseline data for each patient in the study included detailed clinical assessment, physiological studies, high-resolution computed tomography (HRCT), and surgical lung biopsies (SLBs).

### Cells and cell culture condition

Lung fibroblasts were cultured in complete medium at 37 °C and 10% CO_2_. Medium was changed twice a week and cells were passaged when 70–90% confluency was reached. For transcriptomic experiments, 2.5 × 10^5^ lung fibroblasts per well were plated in complete medium into a 6-well plate overnight at 37 °C and 10% CO_2_. Cells were then stimulated with 10 μM CpG (Hycult Biotech) and/or treated with 500 nM Nu7441 (Selleckchem.com) for 24 h (acute) or once every 3 days for a total of 25 days. After treatment, images of the cultured cells were acquired using an EVOS FL inverted microscope at 20x magnification (Thermo Fisher Scientific). Cells were then trypsinized for Flow Cytometry or lysed by Trizol™ (Thermo-Fisher Scientific) for qPCR analysis as described. For studies assessing Collagen 1, IL-8, IL-1ß and αSMA protein expression, cells were then stimulated with 10 μM CpG (Hycult Biotech), 20 ng/ml recombinant TGFß1 (R&D systems) and/or treated with 500 nM of Nu7441 (Selleckchem.com) for 24 or 72 h.

### Immunohistochemistry

Lung tissue was fixed in 10% NBF solution for 24 h and subsequently transferred into tissue cassettes and kept in a 70% ethanol solution for approximately 24 h. Lungs were then processed using routine histology techniques. Sections (4 μm) were deparaffinized, hydrated and subjected to antigen retrieval in 10 mM Citric acid solution (pH 6.0). Samples were then stained with rabbit anti-DNA-PKcs monoclonal antibody (Clone Y393, Abcam) or rabbit anti-CgA polyclonal antibodies (Abcam) overnight at 4 °C and processed as previously described [9]. Images were obtained using Zeiss Axio Observer Z1 microscope and the Zeiss Zen 2012 v 1.1.2.0 software (Zeiss) or ScanScope AT System. The quantification of DNA-PKcs expression (Integrated Optical Density) was performed using Image-Pro Premier software.

### Immunofluorescence analysis

Lung fibroblasts were plated onto Lab-Tek II Chamber Slides (Fisher Scientific) and stimulated with 1 μM biotinylated-CpG (Biotin-CpG; InvivoGen) for 24 h at 37 °C and 10% CO_2_. Cells were then washed with DPBS and fixed with 4% paraformaldehyde solution for 10 min at room temperature. Paraformaldehyde was then washed, and the cells were permeabilized using 0.5% TritonX-100 in DPBS for 10 min at room temperature, washed and blocked using antibody dilution/blocking solution (DPBS + 2% bovine serum albumin + 2% normal goat serum + 0.025% sodium azide) for 30 min at room temperature. Cells were then incubated with rabbit anti-human-DNA-PKcs monoclonal antibody (Clone Y393, Abcam) or IgG control antibody overnight at 4 °C, then washed and incubated with 1:500 diluted Alexa-flour 488-conjugated anti-rabbit (Thermo Fisher Scientific) and 1:500 diluted Alexa-flour 594-conjugated streptavidin (Thermo Fisher Scientific) for 1 hour at room temperature. Cells were then washed three times with DPBS and mounted with ProLong Gold Antifade Mountant with DAPI (Thermo Fisher Scientific). For human lung tissues, paraffin embedded tissues were de-paraffinized, rehydrated and subjected to antigen retrieval in 10 mM Citric acid solution (pH 6.0). Slides were then permeabilized using a 10% methanol solution, blocked and stained using anti-SSEA4 and CgA antibodies (Abcam) overnight at 4 °C. As control, IgG isotype antibodies were utilized on serial sections from the same tissue. The next day, slides were washed three times with DPBS and once with DPBS containing 0.05% Tween-20 (DPBS-T), incubated with Alexa-Flour 594 conjugated anti-mouse and Alexa-Flour 488 conjugated anti-rabbit secondary antibodies (Thermo Fisher Scientific) for 1 h at room temperature. Slides where then washed three times with DPBS and once with DPBS-T and mounted with ProLong Gold Antifade Mountant with DAPI (Thermo Fisher Scientific). A Zeiss Axio Observer Z1 microscope and the Zeiss Zen 2012 v 1.1.2.0 software (Zeiss) were used to obtain representative images and fluorescence intensity was set based on the background fluorescence levels observed in the IgG stained samples.

### Quantitative PCR analysis

Cells were lysed in Trizol™ reagent (Thermo-Fisher Scientific) and RNA was extracted as recommended by the manufacturer. One-microgram of RNA was reverse transcribed into cDNA using superscript II reverse transcriptase (Life technology) as previously described [18]. Complementary DNA (cDNA) was subsequently loaded into a TaqMan plate (Thermo-Fisher Scientific) and gene expression analysis were performed using SYBR or TaqMan master mix (Thermo Fisher Scientific) and *ACTA2* [18], *NRF2* [20] or *HO1* [20] Sybr primers or predesigned primers and probes for human-*COL1A1, COL3A1, FN1, VIM, CDKN1A, CDKN1B, NOX4, CCL2, CXCR4, GAS6, IL1B, IL6, TNF or TNFSF10* (Thermo Fisher Scientific). For mouse lungs, lung tissues were homogenized in Trizol™ solution (Thermo Fisher Scientific) using a microsample homogenizer (Pro Scientific). RNA was extracted, and cDNA was generated as described above. Transcriptomic analysis was performed using *ACTA2* [18], *ENO2* (ENO2F: TCAGGGACTATCCTGTGGT; ENO2R: CATTGGCTGTGAACTTGGA), *GLRA1* (GALR1 F: CCATGAGATCACCACAGAC; GALR1 R: GTCAGGGTGATTCTGATGC) or *NOX4* (Nox4 F: CTCAGTCTTTGACCCTCGG; Nox4 R: GGAGAGCCAGATGAACAGG) SYBR primers or predesigned primers and probes for human-*COL17A1*, *COL3A1*, *VIM*, *CDKN1A* and *CHGA* (Thermo Fisher Scientific). Quantitative PCR analysis was performed using a Viia7 thermocycler (Thermo Fisher Scientific) and the data were analyzed using Data Assist software version 3.01 (Thermo Fisher Scientific).

### Soluble collagen 1, IL-8, IL-1ß and in cell αSMA and ß-tubulin ELISA analysis

Five-thousand lung fibroblasts per well were plated into a 96 well plate, stimulated with 10 μM CpG, 20 ng/ml TGFß and/or treated with 300 nM BIBF1120 (Boehringer Ingelheim) or 500 nM Nu7441 (Selleckchem.com) for 24 and 72 h. After stimulation and/or treatment, conditioned supernatants were collected, and the cells were fixed using 4% paraformaldehyde solution. Collagen-1, IL-8, IL-1ß, αSMA and ß-tubulin proteins were quantified as described under the “Soluble Collagen 1, IL-8, IL-1ß and in cell αSMA and ß-tubulin ELISA analysis” Cells were then analyzed for αSMA protein expression using an in-cell ELISA as previously described [21]. ELISA assays were performed to quantify soluble collagen (as previously described [21]) IL-1ß (R&D systems) and IL-8 proteins (R&D systems) as recommended by the manufacturer.

### Flow cytometry

After Nu7441 treatment, lung fibroblasts were trypsinized for 30–45 s at room temperature. Trypsin was then inhibited by adding complete medium and the cells were spun down at 400 x g for 5 min. Cells were resuspended in DPBS + 2% FBS (FACS buffer) and blocked using human TruStain FcX FC receptor blocking solution (Biolegend) for 15 min on ice. Cells were then stained with anti-SSEA4-APC antibodies (Biolegend) for 15 min at 4 °C, then washed twice with FACS buffer and fixed with 5% neutral buffered formalin solution. Flow cytometric data were acquired using a MACSQuant analyzer 10 (Miltenyi Biotech) and the data were analyzed using FlowJo version 10.3 (Flowjo, LLC).

### Invasive wound healing analysis

Invasive wound healing assay was performed as previously detailed [21]. Briefly, 96 well ImageLock™ plates (Essen BioScience) were pre-coated with Basement membrane extract (BME, Trevigen, 50 μg/mL) and maintained at room temperature for at least 1 h. Next, BME solution was removed from the plates and fibroblasts were plated (3.5 × 10^4^/well) and incubated overnight at 37 °C and 10% CO_2_. The wells containing cells were scratched using the WoundMaker™ (Essen BioScience), washed with PBS and then layered with 4 mg/ml Matrigel containing vehicle or 500 nM Nu7441. Cells were monitored using an IncuCyte ZOOM live cell imager (Essen Biosciences) at 37 °C, 10% CO_2_ for 150 h. Wound closure was measured using the IncuCyte ZOOM Software (Essen Biosciences) as relative wound density [measurement of the spatial cell density in the wounded area relative to the area outside the wounded area over time]. 0% represents the wound when there are no cells in the scratched site at t = 0 and 100% represents a wound density where the cell density in the wound is equivalent to the density outside the wound, i.e. 100% wound healing.

### Bioinformatic analysis of publicly available datasets

Previously analyzed publicly available data sets (GSE103488 [9]) from FACS sorted SSEA4^+^ versus non-sorted SSEA4^−^ cells were re-analyzed as follows: IPF samples utilized in these datasets were divided based on rate of progression, slow progressing IPF (slow-IPF; S161, S109, S170 & S76A; *n* = 4) or rapid progressing IPF (rapid-IPF; defined as described herein and previous study [18]; S99, S148, S48, S69, S56 & S180; *n* = 6), and fold changes were calculated relative to normal cells (NL4, NL7 & NL16; *n* = 3) and the data were analyzed using Ingenuity integrated pathway analysis (IPA; QIAGEN Inc., https://www.qiagenbioinformatics.com/ products/ingenuity- pathway-analysis). Stacked bar graphs of enriched ingenuity canonical pathways (depicting the percentage of transcripts that were upregulated and down regulated) were set to show all pathways with a minimum -log(*p*-value) 3.0 or higher. Stacked bar graphs were exported and are depicted in the figures.

### Mice

Eight-Ten-week-old, female, pathogen free NOD Cg-Prkdc^SCID^ IL2rg^Tm1wil^Szi (NSG) were ordered from Jackson Laboratories. Mice were housed in disposable plastic cages containing Sani-Chip bedding (*n* = 4–5 mice per cage) and allowed to acclimate for a minimum of 7 days prior to utilization. To model the loss of DNA-PKcs activity in xenografted IPF cells in NSG mice (20–25 g), IPF lung explant cells was intravenously administered as previously described [17]. Briefly, mice were physically restrained and 1 × 10^6^ IPF lung explant cell (0.25 mL) was intravenously injected into the tail vein of each mouse (n = 4–5 per group). Thirty-five days after administration, humanized mice were interperitoneally treated with saline or 10 mg/kg of Nu7441 solution (dissolved at 0.5 mg/ml in DPBS and sonicated in a water bath sonicator), twice a week for a total of 4 weeks. After a total of 7 weeks (63 days), mice were euthanized by inhalational isoflurane anesthesia overdose, followed by cervical dislocation. Lung tissues were collected for hydroxyproline, protein, and transcriptomic analysis. The mass in the thoracic cavity was micro dissected, fixed and paraffin embedded for histological analysis. Animals were randomly assigned to the experimental groups and experiments were performed once.

### Hydroxyproline assay

Total lung hydroxyproline was quantified and analyzed as previously described [17].

### Western blot analysis

Mouse lung tissues were homogenized in DPBS containing protease inhibitors (Thermo Fisher Scientific) using a microsample homogenizer (Pro Scientific). Total protein content was quantified using a DC Protein assay (Bio-Rad Laboratories, Inc.) and equal amount of protein was prepared and loaded onto an 4–15% NuPAGE Bis-Tris Protein Gel as recommended by the manufacturer (Thermo-Fisher Scientific). Gels were transferred using an iBlot Dry blotting system onto nitro-cellulose membranes (Thermo-Fisher Scientific) and the transferred samples were blocked for 30 min at room temperature in 5% non-fat-dry-milk in tris-buffered saline (TBS). Blots were then washed twice and incubated with anti-alpha-smooth muscle actin antibodies (Sigma-Aldrich) at 4 °C overnight. The next day, the blots were washed three times with 0.05% tween-20 containing TBS solution (TBS-T), incubated with a secondary antibody, washed three times after incubation and developed using a chemiluminescent substrate (Thermo-Fisher Scientific). Images of chemiluminescent bands were acquired using a Bio-Rad Gel documentation system (Bio-Rad Laboratories, Inc.). The blots were then washed in TBS-T, blotted with anti-tubulin antibodies (Abcam) and developed in a similar manner. Densitometric analysis were performed using Image Lab Software version 6.0 (Bio-Rad Laboratories, Inc.).

### Histologic analysis

Left lung tissue was collected and processed as previously described [17]. Images were obtained using Zeiss Axio Observer Z1 microscope and the Zeiss Zen 2012 v 1.1.2.0 software (Zeiss) or ScanScope AT System.

### Study approval

All experiments with primary human lung tissues and cell lines derived from these tissues were approved by an IRB at Cedars-Sinai Medical Center and University of Michigan (Approval numbers: Pro00034067 and Pro00044849). Moreover, all patients were informed and written consent to participate was obtained prior to inclusion in the studies. All animal procedures followed the guidelines of the Animal Welfare Act and the Public Health Policy on Humane Care and Use of Laboratory Animals of the United Sates and were approved by the Institutional Animal Care and Use Committee at Cedars-Sinai Medical Center (IACUC005136).

### Statistical analysis

All statistical analyses were performed using GraphPad Prism software version 7 (GraphPad). Data were expressed as means ± SEM and assessed for significance by One-way ANOVA followed by Tukey’s multiple comparisons test or Mann-Whitney two-tailed non-parametric test, as detailed in the figure legends. *P* < 0.05 were considered statistically significant.

## Results

### Loss of DNA-PKcs protein in IPF lung tissues

**Fig. 1:**
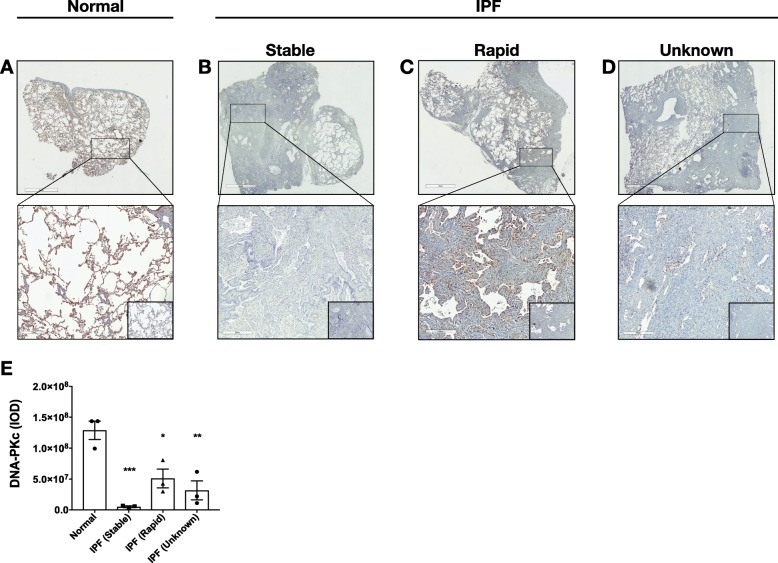
DNA-PKcs expression is reduced in IPF lung tissues. **a**-**d** Shown are representative images of normal (**a**) and IPF (b-d) lung explants from patients with stable (**b**), rapid (**c**), and unknown (**d**) progression of disease stained with anti-DNA-PKcs antibodies or IgG control (inlay) antibodies. **e** Integrated Optical Density (IOD) of DNA-PKcs IHC staining was quantified using Image-Pro Premier. Groups and scale bars are as indicated on the images. *n* = 3 normal, *n* = 3 Stable IPF, *n* = 3 Rapid IPF, and *n* = 3 Unknown IPF. *p ≤ 0.05, **p ≤ 0.01, and ***p ≤ 0.005 compared to normal lung explants. One-way ANOVA followed by Tukey

Recently, we have reported that loss of clusterin protein was associated with reduced DNA repair capacity and disrepair in IPF and mouse lung-epithelial cells [9]. Further, a recent report has shown that clusterin protein expression is also down-regulated in IPF lung fibroblasts [22]. In the present study, DNA-PKcs expression was assessed in lung tissues from normal and IPF patients with stable, rapid, and unknown progression of disease by immunohistochemical analysis. Albeit a relatively low number of patient samples were assessed (*n* = 3 per group), quantification revealed that DNA-PKcs was significantly decreased in IPF lungs compared to normal lungs (Fig. 1a-e). No significant difference was observed in DNA-PKcs expression between the stable, rapid, and unknown progression IPF groups (Fig. 1a-e), suggesting that DNA-PKcs expression is reduced in IPF compared with normal, regardless of disease progression.

### DNA-PKcs is not required for innate DNA sensing of hypomethylated CpG DNA by normal and IPF lung fibroblasts

**Fig. 2:**
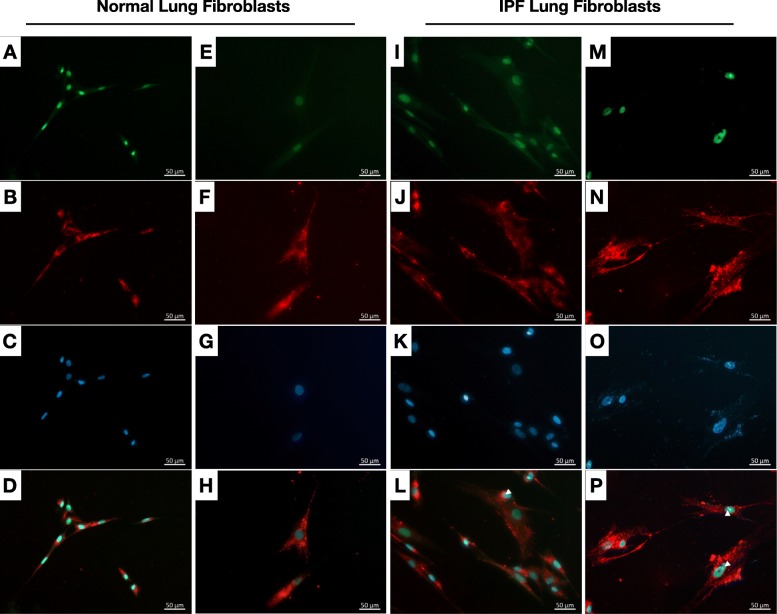
DNA-PKcs does not co-localize with hypomethylated DNA in normal and IPF lung fibroblasts. **a**-**p** Normal and IPF lung fibroblasts were treated with 1 μM biotinylated-hypomethylated CpG DNA (biotin-CpG) for 24 h and then fixed and stained with Alexa-flour 594-conjugated-strepdavidin and anti-DNA-PKcs followed by Alexa-flour 488 conjugated secondary antibodies. As a control, untreated cells were stained with Alexa-flour 594-conjugated-strepdavidin and IgG control antibodies. Shown are representative images acquired at 200x magnification showing the localization of DNA-PKcs (**a**, **e**, **i** & **m**, green), biotin-CpG (**b**, **f**, **j** & **n**, red) or DAPI stained nuclei (**c**, **g**, **k** & **o**, blue) in normal (**a**-**h**) and IPF (**i**-**p**) lung fibroblasts. Composite images are shown in panels **d**, **h**, **l** & **p** for normal and IPF lung fibroblasts, respectively. *n* = 2 normal and *n* = 2 IPF. Scale bars = 50 μm

**Fig. 3:**
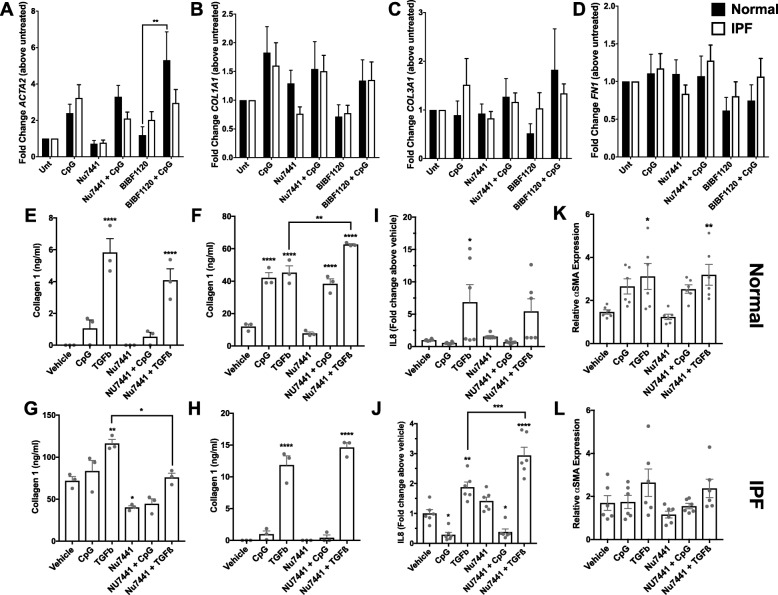
DNA-PKcs kinase activity is not required for innate DNA sensing by lung fibroblasts. **a**-**d** Lung fibroblasts were stimulated with 10 μM of hypomethylated CpG DNA (CpG) and/or treated with 500 nM Nu7441 or 300 nM BIBF-1120 for 24 h. After 24 h, RNA was extracted and transcriptomic analysis for myofibroblasts markers were performed. Shown is the average fold change of *ACTA2* (**a**)*, COL1A1* (**b**)*, COL3A1* (**c**) & *FN1* (**d**) transcript expression in CpG stimulated and/or Nu7441 or BIBF-1120 treated relative to untreated lung fibroblasts. *n* = 3 normal; *n* = 5 IPF. Data shown are mean ± s.e.m.; **p ≤ 0.01 as indicated by the bar. 2-way ANOVA followed by Tukey’s multiple comparisons test. **e**-**h** Lung fibroblasts were stimulated with 10 μM CpG or 20 ng/ml TGF-ß1 and/or treated with 500 nM Nu7441 for 72 h. Shown is the average concentration of secreted collagen 1 protein by two normal (**e**-**f**) and two IPF (**g**-**h**) lung fibroblasts treated in triplicate. Data shown are mean ± s.e.m. *p ≤ 0.05, **p ≤ 0.01, and ****p ≤ 0.0001 compared to vehicle group or as indicated by the bars. One-way ANOVA followed by Tukey’s multiple comparisons test. **i**-**j** Shown is the average fold change of IL-8 protein, secreted by normal (**i**) and IPF (**j**) lung fibroblasts 72 h after stimulation and/or Nu7441 treatment relative to vehicle treated cells. **k-l** Seventy-two hours after stimulation and/or treatment, cells were fixed, permeabilized and stained with anti-αSMA and ß-tubulin antibodies. Shown is the average ß-tubulin normalized expression of αSMA protein in stimulated and Nu7441 treated normal (**k**) or IPF (**l**) fibroblasts relative to their respective vehicle treated groups. **i**-**l** Data shown are mean ± s.e.m. *n* = 2 normal and *n* = 2 IPF treated in triplicate. *p ≤ 0.05, **p ≤ 0.01, ***p ≤ 0.001, and ****p ≤ 0.0001 compared to vehicle group or as indicated by the bar. One-way ANOVA followed by Tukey’s multiple comparisons test

Lung fibroblasts differentiate into myofibroblasts in response to hypomethylated CpG DNA (CpG; [18]) stimulation. Further, several reports have suggested that DNA-PKcs is activated in response to CpG stimulation in immune and non-immune cells, where it promotes type I interferon, IL-6, IL-12 and/or IL-10 expression [23–25]. With this background, we next determined whether DNA-PKcs mediated CpG-induced myofibroblast differentiation in primary lung fibroblasts. Immunofluorescence analysis of cells stimulated with 1 μM of biotinylated-CpG showed that CpG was predominantly localized in focal cytosolic and perinuclear regions in normal and IPF lung fibroblasts (Figs. 2b-d, f-h, j-l and 3n-p) after 24 h. DNA-PKcs was localized in the nucleus of normal and IPF cells (Fig. 2a, e, i & m) at this time, but this protein rarely co-localized with biotinylated CpG DNA in normal cells (Fig. 2a-h) and occasionally co-localized with nuclear biotinylated-CpG DNA in IPF cells (Fig. 2i-p, white arrowheads).

To determine the role of this kinase in CpG-induced myofibroblast differentiation, normal and IPF lung fibroblasts were activated with 10 μM of CpG and treated with 500 nM of Nu7441 (a specific DNA-PKcs small molecule inhibitor [16]) or 300 nM of BIBF-1120 (a multi-receptor tyrosine kinase inhibitor, also known as nintedanib and OFEV) for 24 h. Transcriptomic analysis for myofibroblast markers in these treated cells revealed that CpG induced an increase of *ACTA2* and *COL1A1* transcript expression (Fig. 3a-b) and did not induce any significant changes in *COL3A1* and *FN1* transcripts (Fig. 3c-d) in normal and IPF lung fibroblasts. However, neither Nu7441 or BIBF-1120 treatment significantly reduced CpG-induced *ACTA2* or *COL1A1* transcript expression in normal or IPF lung fibroblasts (Fig. 3a-b). In separate experiments, cells were stimulated with 10 μM CpG or 20 ng/ml of TGFß1 in the presence or absence of 500 nM Nu7441 for 24 or 72 h followed by αSMA, Collagen 1, IL-8 or IL-1ß protein quantification. Seventy-two hours after stimulation, CpG induced a significant increase in secreted collagen 1 protein from one normal lung fibroblast line (Fig. 3f) but did not consistently modulate the secretion of this matrix protein from the other fibroblast lines (Fig. 3e & g-h, respectively). TGFß1 stimulation significantly increased the concentration of secreted collagen 1 in all four fibroblast lines assessed (Fig. 3e-h). Consistent with the transcriptomic analysis, Nu7441 treatment did not modulate CpG-induced collagen 1 secretion (Fig. 3f) and did not consistently modulate TGFß1-induced collagen 1 secretion, where this inhibitor enhanced TGFß1-induced collagen 1 secretion in one normal fibroblast line (Fig. 3f) and inhibited TGFß1-induced collagen 1 secretion in one IPF fibroblast line (Fig. 3g). Seventy-two hours after treatment, neither CpG nor TGFß1 induced consistent IL-8 protein expression and/or secretion by normal lung fibroblasts (Fig. 3i), however, TGFß1 promoted IL-8 protein expression and/or secretion by IPF lung fibroblasts, and this effect was enhanced by Nu7441 treatment (Fig. 3j). Further, TGFß1 and, to a lesser extent, CpG, stimulation induced a modest and Nu7441- insensitive elevation of αSMA protein expression in normal but not IPF lung fibroblasts (Fig. 3k-l). Finally, neither activators nor Nu7441 treatment induced any consistent changes in IL-1ß protein expression and/or secretion at 24 h after IPF lung fibroblast treatment and/or stimulation (Additional file 1: Figure S1A-B). Collectively, these results suggest that DNA-PKcs does not regulate CpG-induced lung fibroblasts myofibroblast differentiation but may be involved in senescence.

### Chronic inhibition of DNA-PKcs kinase activity leads to an expansion of SSEA4^+^ mesenchymal progenitor cells and the expression of myofibroblast, senescence-associated and inflammatory transcripts by lung fibroblasts

**Fig. 4:**
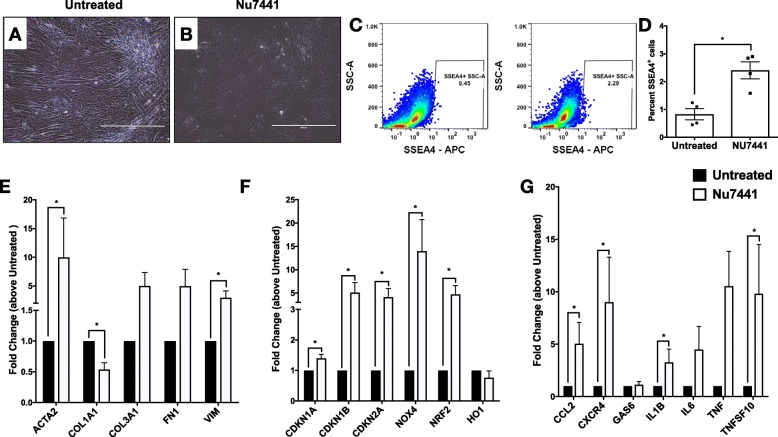
Loss of DNA-PKcs kinase activity promotes the expansion SSEA4^+^ progenitor cells and senescence-associated transcripts. Lung fibroblasts were cultured and treated with 500 nM of Nu7441 every 3 days for a total of 25 days. As a control, untreated fibroblasts were utilized. **a**-**b** Shown are representative images of untreated (**a**) or 25-day Nu7441 treated (**b**) lung fibroblasts. **c** After 25 days, lung fibroblasts were trypsinized and stained with anti-SSEA4 antibodies. Shown are representative dot plots depicting SSEA4^+^ cells within untreated (C, left) and 25-day Nu7441 treated (C, right) fibroblast cultures. **d** Shown is the mean percentage of SSEA4^+^ cells in untreated and 25-day Nu7441 treated fibroblast cultures. Data shown are mean ± s.e.m.; *n* = 4. *p ≤ 0.05 via Mann-Whitney two-tailed non-parametric test. **e**-**g** Twenty-five days after culture with or without Nu7441, RNA was extracted from lung fibroblasts and qPCR analysis was performed for myofibroblast (**e**), senescence and stress-associated (**f**) and inflammatory (**g**) transcripts. Depicted is the average fold change of expression of Nu7441 relative to vehicle treated lung fibroblasts. *n* = 4; *p ≤ 0.05 via Mann-Whitney two-tailed non-parametric test

To further explore the persistent inhibition of DNA-PKcs in human lung fibroblasts, cells were treated with 500 nM of Nu7441 once every 3 days for a total of 25 days, after which cells were analyzed via flow cytometry and transcriptomic analysis. Untreated cells were utilized as controls. At day 25 after Nu7441 treatment, lung fibroblasts were morphologically distinct compared to untreated cells (Fig. 4a-b), most notably cells treated with the inhibitor were more hyperplastic relative to their untreated counterparts. Flow cytometric analysis showed that there was a significant change in the percentage of SSEA4^+^ progenitor cells in Nu7441-treated cultures compared with untreated cultures (Fig. 4c-d). Transcriptomic analysis of Nu7441-treated and untreated fibroblasts showed that this treatment caused a significant increase in the myofibroblast-associated *ACTA2* & *VIM* (Fig. 4e)*,* the senescence-associated *CDKN1A, CDKN1B, CDKN2A, NOX4* (Fig. 4f), and the stress-response and pro-inflammatory transcripts *NRF2, CCL2, CXCR4, IL1B* and *TNFSF10* (Fig. 4g) compared with untreated cells. Surprisingly, Nu7441 treatment significantly reduced *COL1A1* transcript relative to untreated fibroblasts (Fig. 4e). We then assessed the modulation of lung fibroblast invasive wound healing by Nu7441. Treatment of lung fibroblasts with 500 nM of Nu7441 over 150 h reduced the invasive wound healing in both normal (Additional file 2: Figure S2A-C) and IPF lung fibroblast lines (Additional file 2: Figure S2D-E). Together, these results demonstrate that inhibition of DNA-PKcs activity enriched for SSEA4^+^ mesenchymal progenitor cells, reduced lung fibroblast invasion, and promoted senescence-associated markers in vitro.

### Loss of *PRKDC* transcript expression and various components involved in DNA damage response in SSEA4^+^ mesenchymal progenitor and SSEA4^−^ mesenchymal progeny cells

**Fig. 5:**
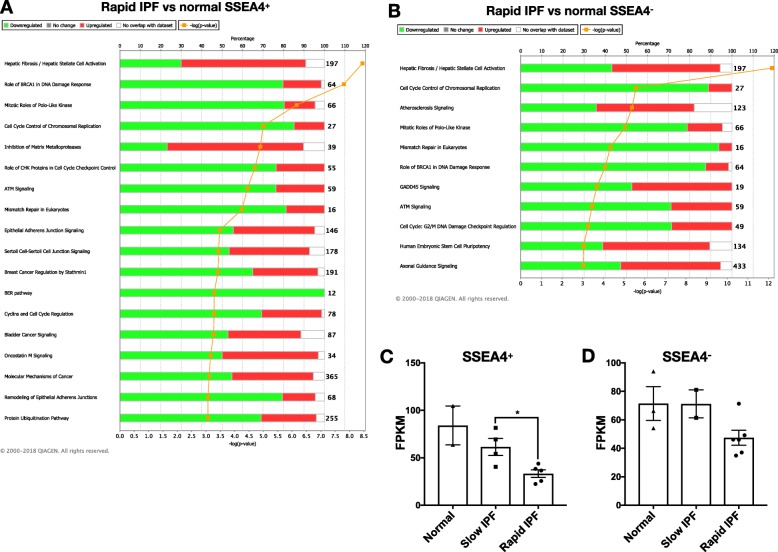
Loss of *PRKDC* transcript and DNA damage response components in SSEA4^+^ and SSEA4^−^ IPF fibroblasts. SSEA4^+^ cells were sorted from normal and IPF lung fibroblast cultures. RNA was extracted from the sorted cells SSEA4^+^ and non-sorted SSEA4^−^ cells and subject to RNA sequencing analysis as previously described (GSE103488 [9]). **a**-**b** Shown are Ingenuity canonical pathway analysis of Rapid-IPF versus normal SSEA4^+^ cells (**a**) and Rapid-IPF versus normal SSEA4^−^ cells (**b**). Ingenuity was set to consider transcripts with an FPKM value ≥0.2 and a fold change ≥1.5 & ≤ − 1.5 (**a**) and FPKM value ≥1 and a fold change ≥1.5 & ≤ − 1.5 (**b**). Percentage depicts the proportion of transcripts from the transcriptomic analysis that are annotated in the Ingenuity canonical pathway. The percentage of transcripts that are downregulated or upregulated in each canonical pathway are depicted in green or red, respectively. **c**-**d** Shown are the mean FPKM counts for *PRKDC* in normal, slow-IPF and rapid-IPF SSEA4^+^ progenitors (**a**) and SSEA4^−^ fibroblasts (**b**). Data shown are mean ± s.e.m.; *n* = 2–5/group. *p ≤ 0.05 via Mann-Whitney two-tailed non-parametric test

Next, we assessed the expression of proteins involved in DNA repair in normal and IPF lung fibroblasts and mesenchymal progenitor cells by reanalyzing previously published transcriptomic datasets (GSE103488 [9]) from sorted normal and IPF SSEA4^+^ mesenchymal progenitor cells [6, 26] and SSEA4^−^ fibroblasts based on IPF progression. The IPF datasets were grouped by progression as follows: slow progressing IPF (slow-IPF; S161, S109, S170 & S76A) versus rapid progressing IPF (rapid-IPF; defined in the methods section and as previously described [18]; S99, S148, S48, S69, S56 & S180). Fold-changes were then calculated for each group compared with normal cells and the data were analyzed using Ingenuity Integrated Pathway Analysis (IPA). As summarized in Fig. 5, a loss of transcripts encoding for components of various DNA damage response and repair proteins were observed in both rapid-IPF SSEA4^+^ (Fig. 5a) and SSEA4^−^ cells (i.e. progeny) (Fig. 5b), including role of BRCA1 in DNA damage response, ATM signaling, mismatch repair in eukaryotes, BER pathway and Cell cycle: G2/M DNA damage checkpoint regulation pathways compared with the normal mesenchymal progenitors and progeny. However, these pathways were not present following a comparison of SSEA4^+^ and SSEA4^−^ cells from slow progressing IPF patients with normal mesenchymal cell types (Additional file 3: Figure S3A-B). Because of the loss of various DNA repair pathways in progenitor and mesenchymal cells, we next assessed the expression of *PRKDC* (encoding for DNA-PKcs), which is required for DNA damage signaling, double strand break repair, telomere maintenance and resistance to oxidative stress [11, 12, 27, 28] in rapid- and slow-IPF SSEA4^+^ progenitors and SSEA4^−^ fibroblasts. Mining of transcriptomic datasets showed that there was a loss *PRKDC* transcript in cells derived from rapid-IPF fibroblast cultures (Fig. 5c-d). Together, these results suggest that cultured SSEA4^+^ mesenchymal progenitor and SSEA4^−^ mesenchymal progeny cells from progressive IPF patients were characterized by a loss of PRKDC transcript expression and various components involved in DNA damage response and/or DNA damage repair pathways.

### CgA protein is highly expressed by SSEA4^+^ mesenchymal progenitor cells and in IPF lung tissues

**Fig. 6:**
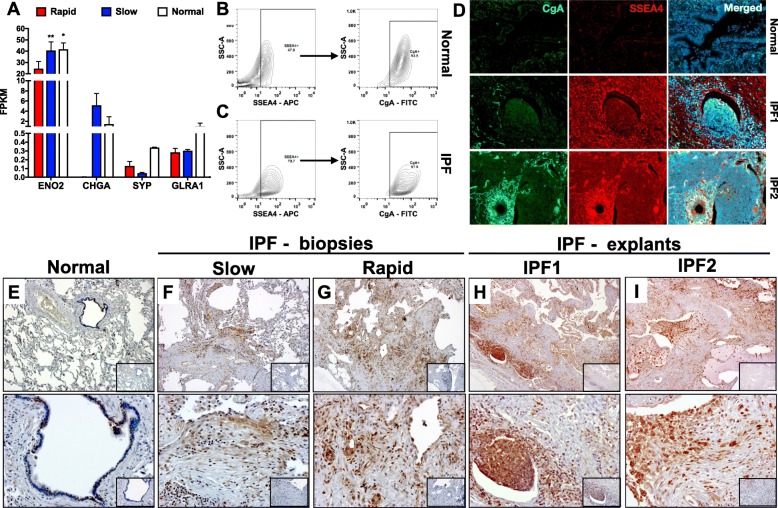
Abundance of CgA expression in SSEA4^+^ progenitor cells and IPF lung tissues. **a** SSEA4^+^ cell RNAseq datasets (GSE103488 [9]) were mined for the expression of various lineage transcripts. Shown are the average FPKM values from 2 Normal-, 4 slow- and 4 rapid-SSEA4^+^ cells. **b**-**c** Normal and IPF stromal cultures were permeabilized and stained SSEA4 and CgA followed by flow cytometric analysis. Representative flow cytometric contour plots derived from 3 normal (**b**) and 2 IPF (**c**) stromal cultures stained and gated for SSEA4 (left) showing the expression of CgA protein in the gated cells (right). **d** Normal or IPF lung explants were stained for CgA and SSEA4 followed by fluorescently conjugated secondary antibody and fluorescent microscopy analysis. Representative images from Normal (top) and two IPF patients (bottom two rows) are shown stained with CgA (left) and SSEA4 (middle) and the merged composite (right) acquired at 200x magnification. **e**-**i** Normal (**e**), slow- (**f**), rapid- (**g**), IPF biopsies or explants (**h**-**i**) were histologically stained for CgA. Representative images were taken from 3 normal, 4 slow- & 5 rapid-IPF biopsies and 12 IPF explants. IgG control staining are shown in inlayed images

Given the expansion of SSEA4^+^ cells in fibroblast cultures that were treated chronically with Nu7441, further characterization of these cells was performed. Several reports have suggested that SSEA4^+^ cells are mesenchymal progenitor cells [6, 26] and we have recently observed that these cells are also enriched with basal-epithelial cell transcripts [9]. Mining of transcriptomic datasets (GSE103488 [9]) for additional airway epithelial cell lineage markers showed that these cells expressed various markers of neuroendocrine cells, including *ENO2, CHGA, SYP* and *GLRA1* (Fig. 6a). Further, these markers tended to be significantly enriched in SSEA4^+^ cells from slow-IPF or normal lung fibroblast cultures (Fig. 6a). Consistently, SSEA4^+^ cells from normal and IPF lungs abundantly expressed CgA protein as determined by intracellular flow cytometric analysis (Fig. 6b-c). Rare CgA or SSEA4 immuno-positive cells were detected in normal lung tissues (Fig. 6d, top) but there was an abundance of cells that expressed both CgA and SSEA4 proteins in IPF lungs (Fig. 6d, middle and bottom, Additional file 4: Figure S4). Further, IHC analysis for CgA protein in normal and IPF lung tissues showed that this protein is rarely detected in normal lungs (Fig. 6e) but CgA-positive cells were detected in slow-IPF, rapid-IPF lung biopsies (Fig. 6f-g), and most abundant in end stage lung-explants (Fig. 6h-i). Thus, these results demonstrate that SSEA4^+^ mesenchymal progenitor cells express the neuroendocrine marker, CgA and that there appears to be an expansion of these cells as IPF progresses.

### Inhibition of DNA-PKcs activity promoted the expansion of SSEA4^+^ CgA^+^ cells in humanized NSG mice

**Fig. 7:**
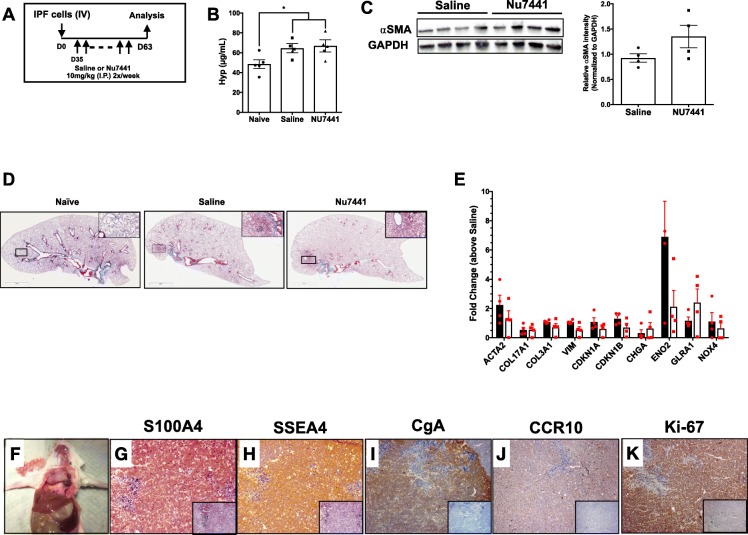
DNA-PKcs inhibition in IPF explant cell-humanized NSG mice promoted expansion of SSEA4^+^ S100A4^+^ cells. **a** IPF lung explant cells were intravenously administered into NSG mice. Thirty-five days after administration, mice were treated interperitoneally with saline or 10 mg/kg of Nu7441, twice a week for 4 weeks. After a total of 63 days, mice were sacrificed, and their lung tissues were collected for analysis. Shown are the (**b**) average hydroxyproline concentration in the lungs of mice and (**c**) representative western blot of lung lysates from saline and Nu7441 treated NSG mice for alpha-SMA (αSMA, top) and GAPDH (bottom) proteins and densitometric ratios of αSMA normalized to GAPDH proteins from saline and Nu7441 treated mice. Data shown are mean ± s.e.m.; n = 4–5/group. One-way ANOVA followed by Tukey’s multiple comparisons test. (**d**) Masson’s trichrome staining of naive and IPF lung explant cell–challenged NSG mouse lungs treated with saline or Nu7441. **e** RNA was extracted from saline or Nu7441 treated NSG mouse lungs and qPCR analysis was performed for various human transcripts. Shown is the average expression of the indicated transcripts in Nu7441 relative to saline treated NSG lungs. Data shown are mean ± s.e.m.; n = 4/group. **f** One out of five mice receiving Nu7441 developed a mass in the thoracic cavity. **g**-**k** Shown are representative images acquired at 50x magnification of the tissue mass stained for human-S100A4 (**g**), SSEA4 (**h**), human-CgA (**i**), human-CCR10 (**j**), Ki-67 (**k**) or IgG control (inlay) antibodies

Our final set of experiments addressed the effects of DNA-PKcs on lung fibrosis in vivo. Recently, we developed a novel humanized NSG model of IPF in which immune and non-immune cells derived from explanted IPF tissue are intravenously administered into NSG mice and induce lung remodeling that is apparent by day 35 after introduction [17]. To study the role of DNA-PKcs in humanized NSG mice, mice were intravenously administered with IPF cells and starting at day 35 after cellular administration, mice were treated interperitoneally with 10 mg/kg of Nu7441 or saline, twice a week for 4 weeks (Fig. 7a). As aforementioned, Nu7441 is a potent and highly selective inhibitor of DNA-PK. In this study, it was used to target injected IPF cells and mimic the loss of DNA-PK observed in IPF. Nu7441 treatment did not modulate hydroxyproline levels in humanized mice relative to the saline-treated groups (Fig. 7b), αSMA protein levels (Fig. 7c), lung remodeling (Fig. 7d) or transcripts encoding for *ACTA2, COL17A1, COL3A1, VIM, CDKN1A, CDKN1B, CHGA, ENO2, GLRA1 and NOX4* (Fig. 7e) (*n* = 4–5 mice per group). However, in 20% of the humanized NSG mice treated with Nu7441, there was a mass in the thoracic cavity (Fig. 7f) (*n* = 1). IHC analysis on tissue extracted from this mass showed that cells within this mass highly expressed human-S100A4 (Fig. 7g), SSEA4 (Fig. 7h), CgA (Fig. 7i), CCR10 (Fig. 7j) and Ki-67 (Fig. 7k) proteins. Collectively, these results suggest that DNA-PKcs may protect against extracellular matrix deposition and the expansion of mesenchymal progenitor cells in vivo.

## Discussion

IPF is a lethal fibrotic lung disease, characterized by excessive ECM deposition leading to the loss of the lung’s architecture and function. This disease is characterized by shortened telomeres in structural cells [3, 29–31], senescence of epithelial progenitor and stromal cells [30, 32–39], microsatellite instability [40, 41] and an increased incidence of lung cancer [42, 43]. However, mechanisms accounting for these features in IPF remain elusive. Given the previously reported role of DNA-PKcs in innate DNA sensing [25, 44], genomic stability, telomere capping and maintenance, cell replication and DNA-damage repair [11, 12], we further assessed both the expression and function of this protein in IPF. In this report, we observed the reduced expression of DNA-PK in the lungs of IPF patients and mesenchymal progenitor cells (SSEA4^+^ cells) and fibroblasts (SSEA4^−^ cells) from IPF patients. Moreover, targeting DNA-PK in vitro revealed the important role of this kinase in regulating senescence in these cell types.

Because DNA-PKcs has been explored in innate DNA sensing [11, 12], studies were first performed to assess the role of this kinase in CpG-mediated fibroblast to myofibroblast differentiation. Immunofluorescence analysis suggested that DNA-PKcs primarily localized in the nucleus of both normal and IPF lung fibroblasts. Twenty-four hours after CpG treatment, this kinase localized in endosomal and peri-nuclear regions with rare localization in the nucleus. Further, we rarely detected colocalization of CpG-DNA and DNA-PKcs, suggesting that this kinase may not act as an innate DNA sensor in lung fibroblasts. Indeed, inhibiting this kinase failed to modulate CpG-induced Collagen 1, α-SMA and IL-1β protein expression in lung fibroblasts. Consistently, the expression of DNA-PKcs transcripts were significantly reduced in SSEA4^+^ mesenchymal progenitor cells and fibroblasts derived from lung tissues of rapidly progressing IPF patients, where we most consistently observe robust CpG mediated myofibroblast differentiation [18]. These results suggest that IPF lung fibroblasts may not utilize DNA-PKcs as an innate DNA sensor.

DNA-PKcs acts as a scaffold and a kinase, where the loss of its kinase activity leads to telomeric aberrations, and radiation sensitization due to reduced NHEJ DNA repair [45–47]. To model the loss of DNA-PKcs in IPF, we chronically treated human lung fibroblast cultures with a highly specific small molecule kinase inhibitor, NU7441 [16]. Chronic treatment of lung fibroblasts with Nu7441 accelerated fibroblast senescence in vitro as evidenced by the increased expression of various senescence markers in these cells. Given the role of DNA-PKcs in DNA repair and checkpoint signaling during replication stress [48, 49], this senescence effect may be due to increased replication stress in the treated cells. This finding is consistent with other studies showing the induction of senescence because of replication stress [50], and suggests that the loss of this kinase may contribute to the abundance of senescent cells in IPF lungs [30, 32–38]. Surprisingly, in in vivo studies, DNA-PKcs inhibition via Nu7441 treatment did not change the expression of senescence markers in the lung tissues. However, these studies were performed in mice lacking DNA-PKcs kinase activity (due to a mutation in the gene encoding for this protein) and thus the lack of any observed effects may be due to the dilution of DNA-PKcs expressing human cells in the mouse lungs. Interestingly, 20% of humanized NSG mice treated with this inhibitor showed a marked expansion in SSEA4^+^ mesenchymal progenitor cells, which were also positive for CgA, S100A4 [26], and CCR10. Further, most of the cells expressed high levels of Ki-67 protein, suggesting a high proliferative index. Given the role of DNA-PKcs in replication stress and its role in preventing telomeric fusions [11, 12], it is possible that targeting this protein in vivo promoted genomic instability and subsequently increased the proliferation of these progenitor cells. However, further studies are required to fully address this possibility since this was observed in only one mouse in the current study.

Since SSEA4^+^ cells were expanded in Nu7441-treated humanized NSG mice, we undertook additional analyses of these cells to determine what other markers these progenitor cells expressed [6, 26]. Surprisingly, this analysis indicated these cells expressed multiple neuroendocrine markers. Indeed, pulmonary neuroendocrine cells were reported to be among the first specialized cell type to appear during lung development [51]. Further, cell-tracing studies have indicated that neuroendocrine cells can give rise to various epithelial cells, including club cells, ciliated cells in response to naphthalene-induced injury [52], which is consistent with the co-expression of various epithelial and neuroendocrine markers in cultured SSEA4^+^ cells. Nevertheless, in addition to epithelial and neuroendocrine markers, SSEA4^+^ cells also expressed stromal markers suggesting that these cells may also give rise to stromal cells. Interestingly, the SSEA4^+^ mesenchymal progenitor cells detected in humanized SCID mice highly expressed the neuroendocrine marker, CgA, and there was an expansion of CgA-expressing cells in IPF lungs. This finding is consistent with a previous report [53], and suggests that there may be aberrant neuroendocrine cells in IPF, and DNA-PKcs regulates the differentiation of SSEA4^+^ mesenchymal progenitor cells. Thus, the loss of DNA-PKcs in IPF might be associated with the emergence of a diverse array of cell types from SSEA4^+^ progenitor cells including those with epithelial cell, neuroendocrine, and/or mesenchymal cell properties.

## Conclusions

In summary, these results demonstrate that loss of DNA-PKcs promotes the expansion of SSEA4^+^ mesenchymal progenitor cells and the senescence of fibroblasts in IPF. Additional studies are warranted to further explore the DNA-PKcs-dependent mechanisms leading to the proliferation of pathologic SSEA4^+^ mesenchymal progenitor cells and the senescence of differentiated mesenchymal cells, and whether these events are modulated by FDA-approved IPF therapeutics including Ofev and Esbriet or other emerging IPF therapeutics.

## Additional files

Additional file 1: **Figure S1.** Inhibition of DNA-PKcs kinase activity does not modulate CpG-mediated IL1ß secretion by IPF lung fibroblasts. Lung fibroblasts were stimulated with 10 μM CpG or 20 ng/ml TGF-ß1 and/or treated with 500 nM Nu7441 for 24 h. (**A-B**) Shown is the average concentration of IL1ß secreted from two senescent IPF lung fibroblasts lines after stimulation and/or Nu7441 treatment in triplicate. (PDF 42 kb)

Additional file 2: **Figure S2.** Inhibition of DNA-PKcs ameliorated lung fibroblast invasive wound healing. Lung fibroblasts were then plated into 96 well plates, scratched using a WoundMaker™ and then layered with 4 mg/ml Matrigel containing vehicle or 500 nM Nu7441. Lung fibroblast invasive wound healing was monitored using an Incucyte Zoom live cell imager. Depicted is a kinetic read-out of wound closure (relative to the initial wound) over 150 h of three normal (A-C) and two IPF (D-E) lung fibroblasts treated in triplicate. (PDF 239 kb)

Additional file 3: **Figure S3.** Ingenuity canonical pathways enriched in Slow-IPF SSEA4^+^ and SSEA4^−^ cells compared to normal cells. SSEA4^+^ cells were sorted from normal and IPF lung fibroblast cultures. RNA was extracted from the sorted cells SSEA4^+^ and non-sorted SSEA4^−^ cells and subject to RNA sequencing analysis as previously described (GSE103488). (**A-B**) Shown are Ingenuity canonical pathway analysis of Slow-IPF versus normal SSEA4^+^ cells (A) and Slow-IPF versus normal SSEA4^−^ cells (B). Ingenuity was set to consider transcripts with an FPKM value ≥0.2 and a fold change ≥1.5 & ≤ − 1.5 (A) and FPKM value ≥1 and a fold change ≥1.5 & ≤ − 1.5 (B). Percentage depicts the proportion of transcripts from the transcriptomic analysis that are annotated in the Ingenuity canonical pathway. The percentage of transcripts that are downregulated or upregulated in each canonical pathway are depicted in green or red, respectively. (PDF 793 kb)

Additional file 4: **Figure S4.** Immunofluorescence IgG control staining of IPF lung tissues. Normal or IPF lung explants were stained IgG antibodies followed by fluorescently conjugated secondary antibodies and microscopy analysis. Representative images from two IPF patients are shown stained with IgG + Alexa Flour 488 conjugated secondary antibody (left), IgG + Alexa Flour 594 conjugated secondary antibody (middle) and the merged composite (right) acquired at 200x magnification. (PDF 405 kb)

## Data Availability

Data and materials will be available for public upon request to the corresponding authors MSH (miriam.hohmann@cshs.org) or CMH (cory.hogaboam@cshs.org).

## Abbreviations

CpG: Hypomethylated CpG DNA
DNA-Pkcs: DNA-dependent protein kinase
ILD: Interstitial lung diseases
IPF: Idiopathic pulmonary fibrosis
SSEA: Stage-specific embryonic antigen
