# Supplementary figures and images for: DNA-PKcs modulates progenitor cell proliferation and fibroblast senescence in idiopathic pulmonary fibrosis
Source: BMC Pulm Med. 2019 Aug 29;19:165. doi: 10.1186/s12890-019-0922-7 (PMC6716822; doi:10.1186/s12890-019-0922-7)

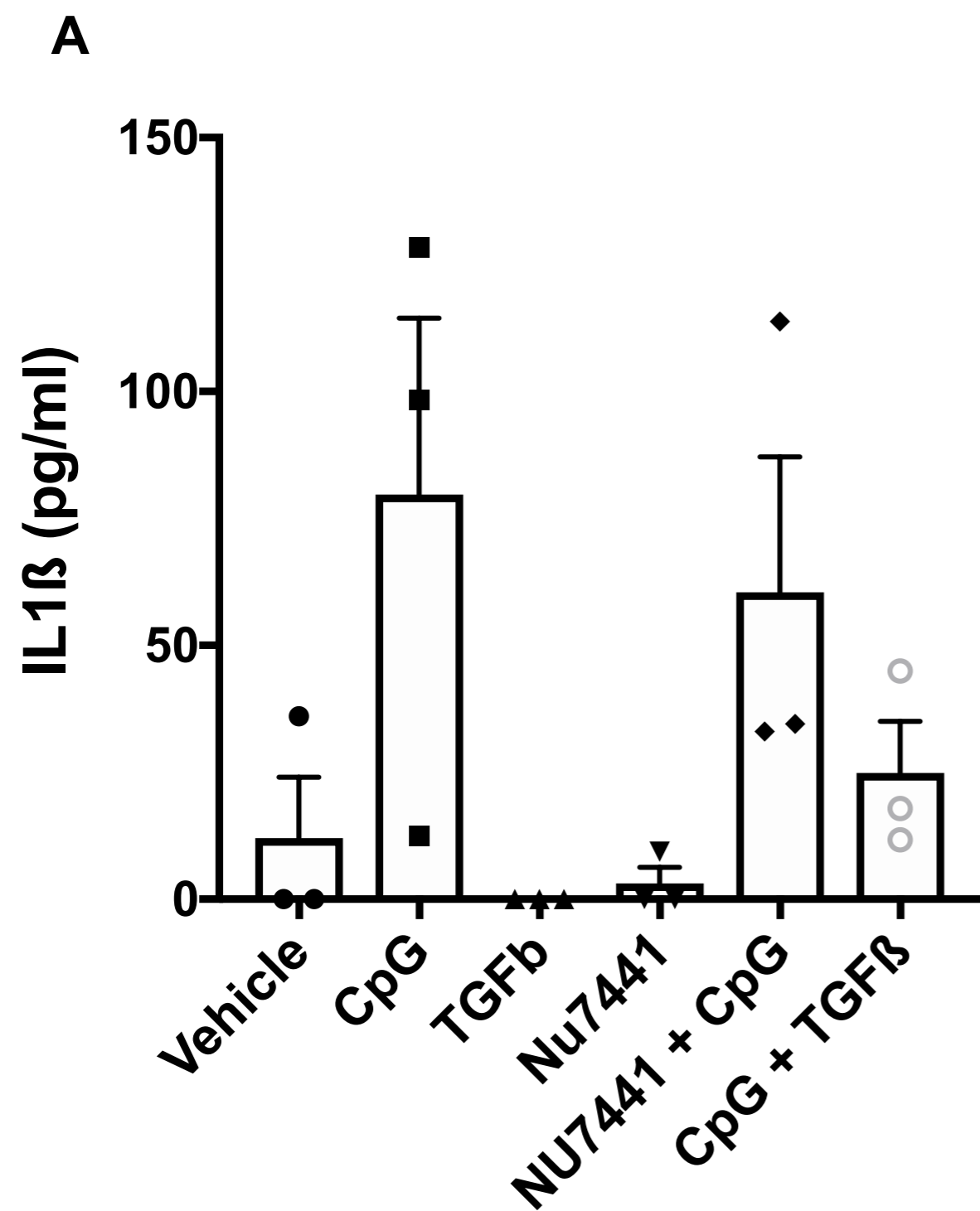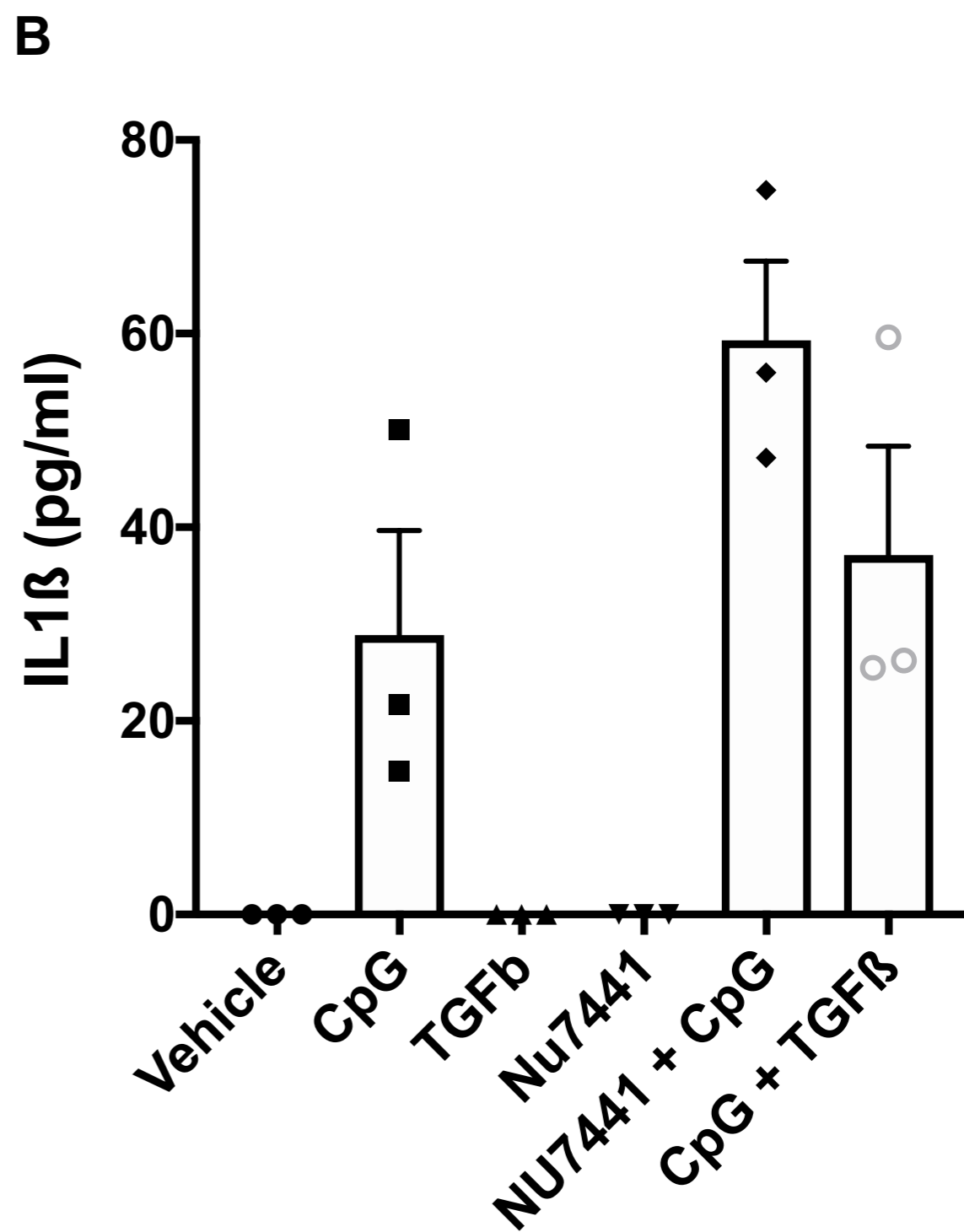

Supplement: Supplementary file 1 — Figure S1. Inhibition of DNA-PKcs kinase activity does not modulate CpG-mediated IL1ß secretion by IPF lung fibroblasts. Lung fibroblasts were stimulated with 10 μM CpG or 20 ng/ml TGF-ß1 and/or treated with 500 nM Nu7441 for 24 h. (A-B) Shown is the average concentration of IL1ß secreted from two senescent IPF lung fibroblasts lines after stimulation and/or Nu7441 treatment in triplicate. (PDF 42 kb) [file 12890_2019_922_MOESM1_ESM.pdf]

**A**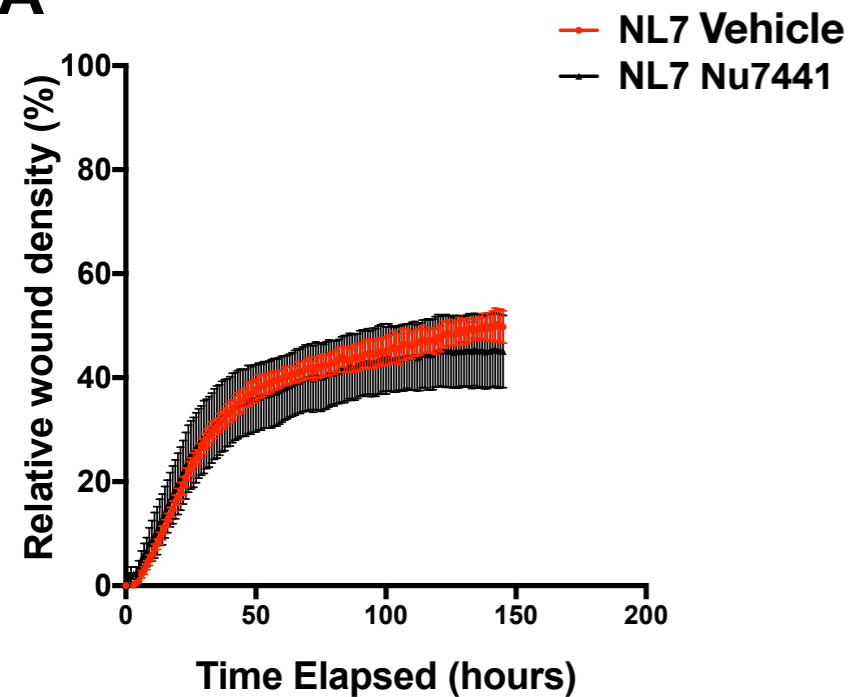**B**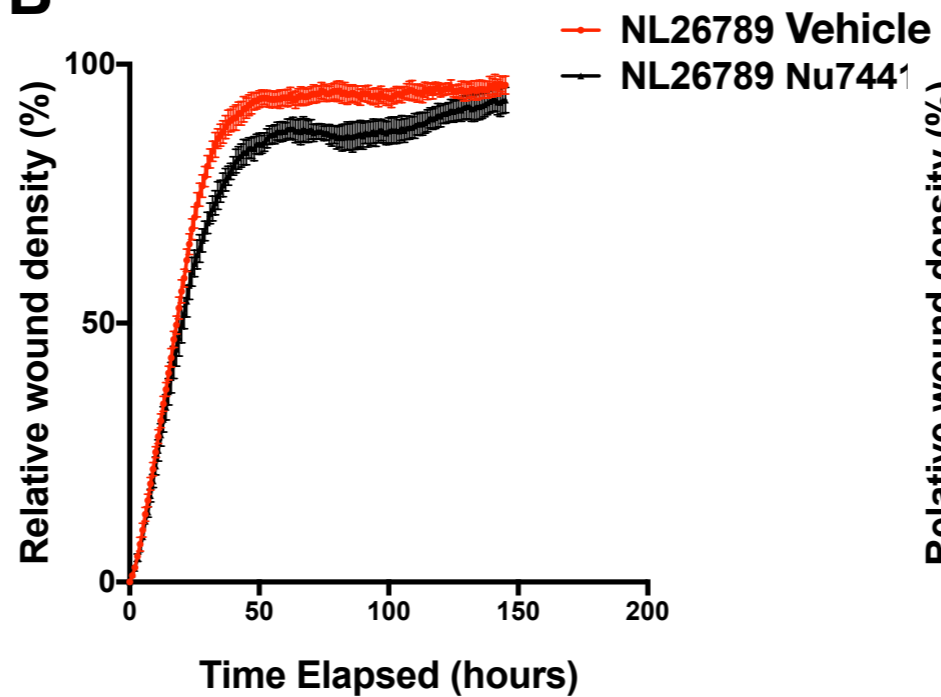**C**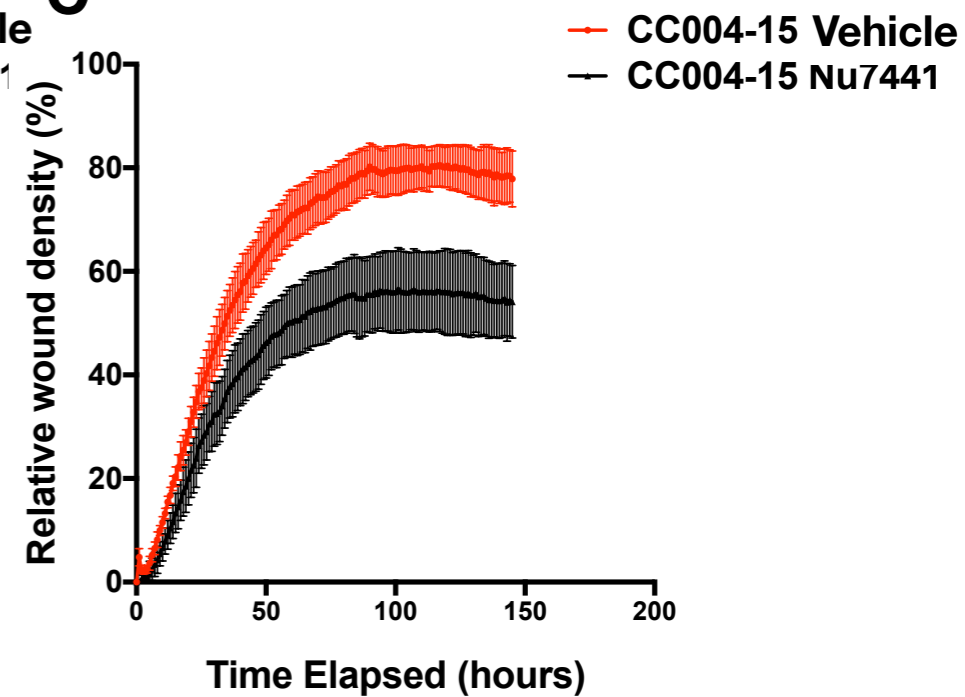**D**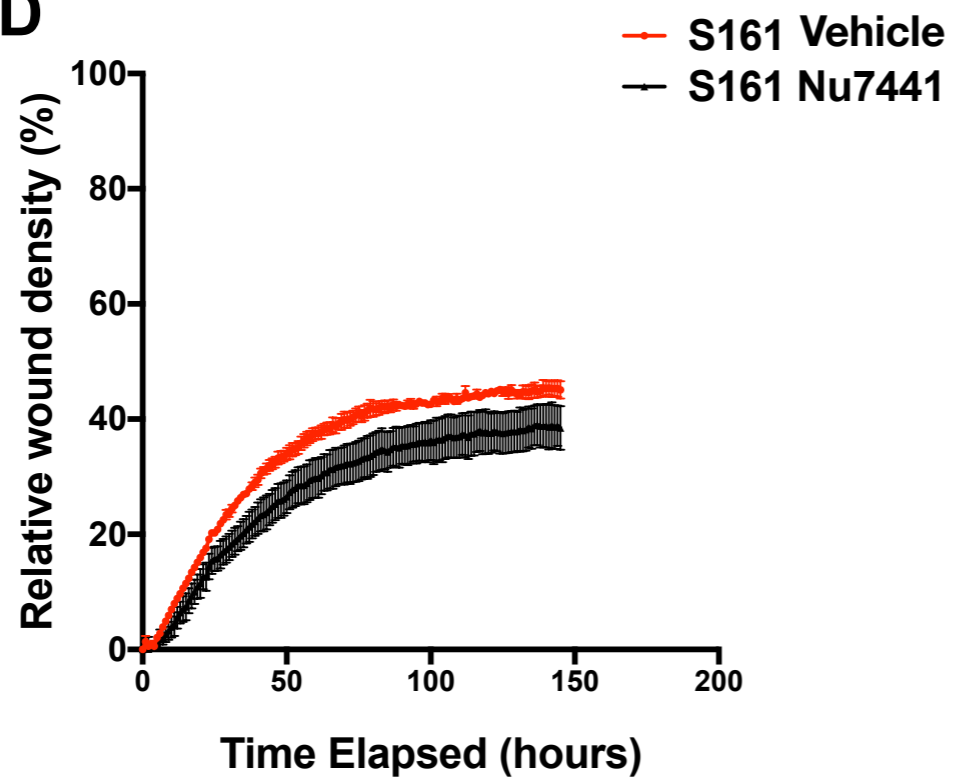**E**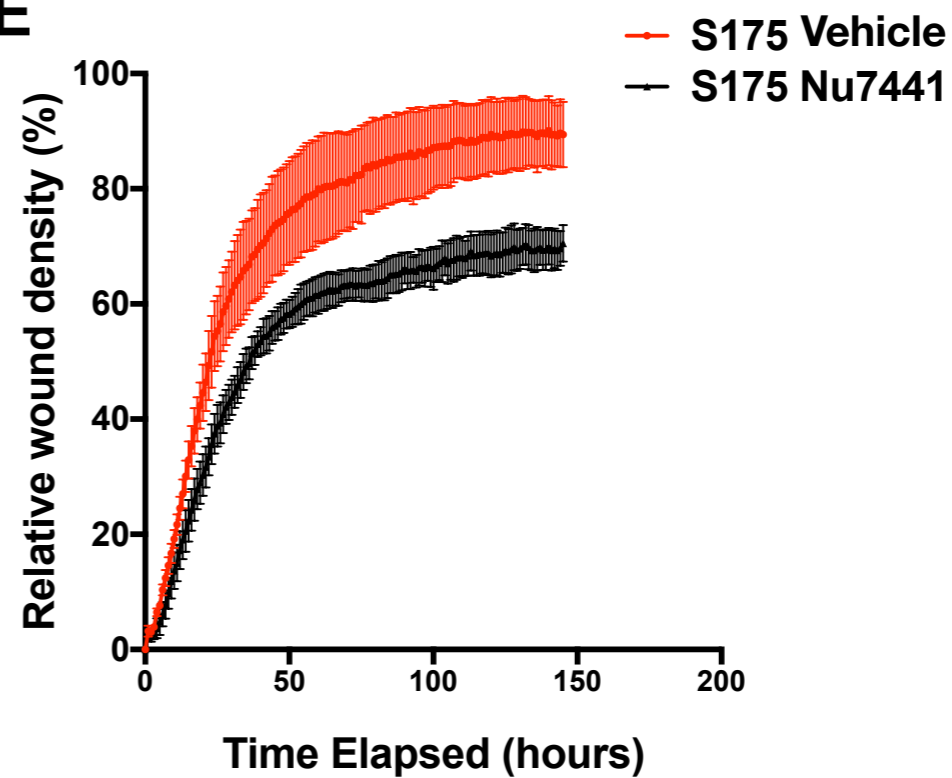

Supplement: Supplementary file 2 — Figure S2. Inhibition of DNA-PKcs ameliorated lung fibroblast invasive wound healing. Lung fibroblasts were then plated into 96 well plates, scratched using a WoundMaker™ and then layered with 4 mg/ml Matrigel containing vehicle or 500 nM Nu7441. Lung fibroblast invasive wound healing was monitored using an Incucyte Zoom live cell imager. Depicted is a kinetic read-out of wound closure (relative to the initial wound) over 150 h of three normal (A-C) and two IPF (D-E) lung fibroblasts treated in triplicate. (PDF 239 kb) [file 12890_2019_922_MOESM2_ESM.pdf]

A

Slow IPF vs normal SSEA4+

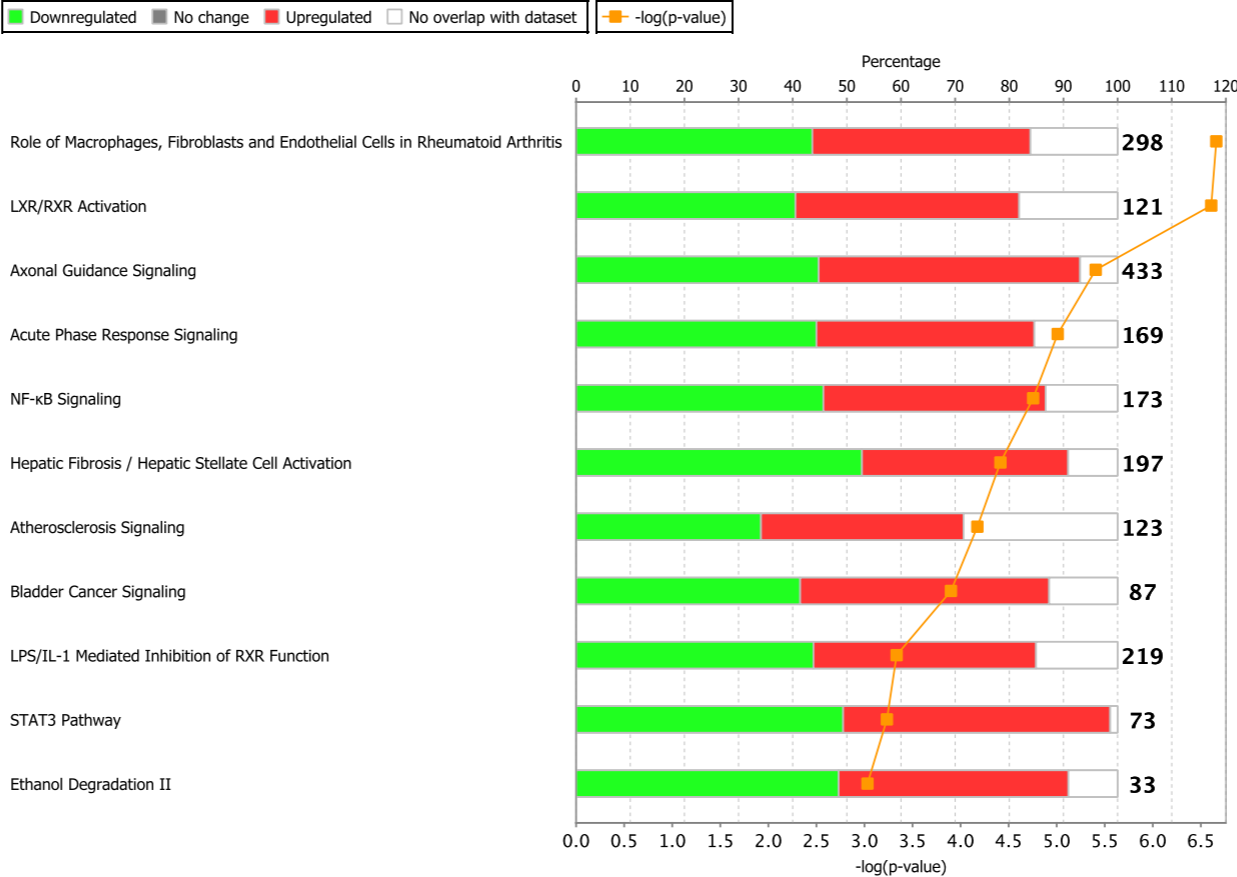

B

Slow IPF vs normal SSEA4-

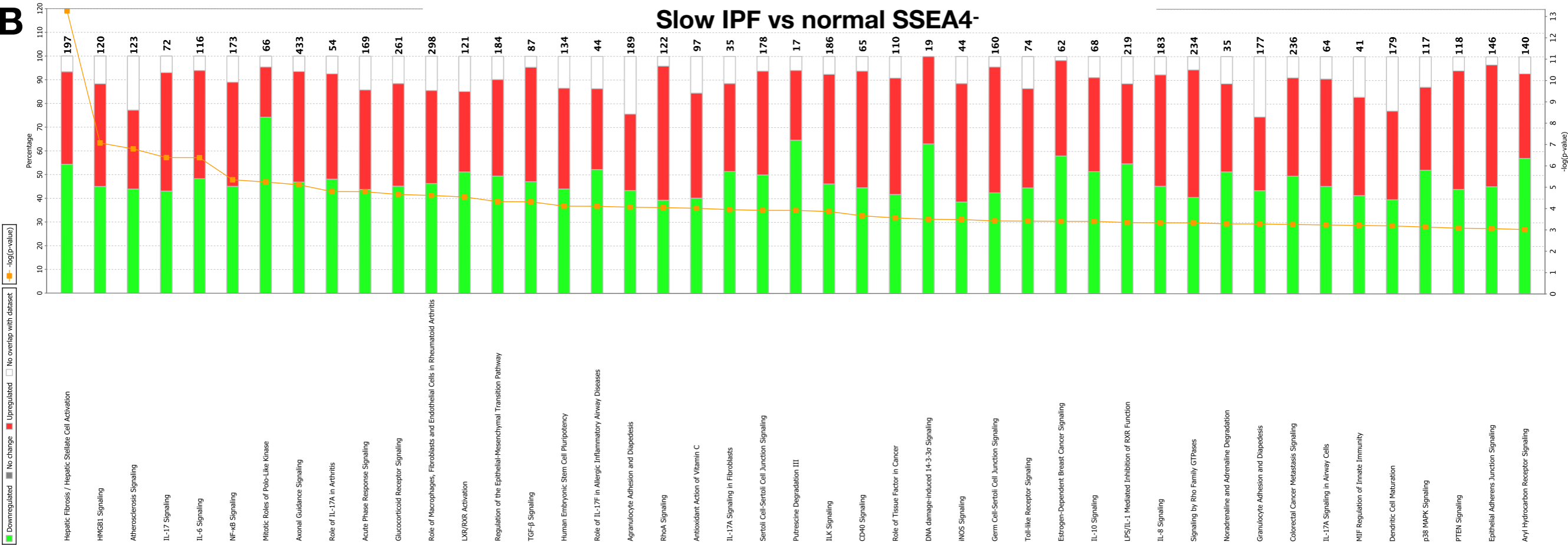

Supplement: Supplementary file 3 — Figure S3. Ingenuity canonical pathways enriched in Slow-IPF SSEA4+ and SSEA4− cells compared to normal cells. SSEA4+ cells were sorted from normal and IPF lung fibroblast cultures. RNA was extracted from the sorted cells SSEA4+ and non-sorted SSEA4− cells and subject to RNA sequencing analysis as previously described (GSE103488). (A-B) Shown are Ingenuity canonical pathway analysis of Slow-IPF versus normal SSEA4+ cells (A) and Slow-IPF versus normal SSEA4− cells (B). Ingenuity was set to consider transcripts with an FPKM value ≥0.2 and a fold change ≥1.5 & ≤ − 1.5 (A) and FPKM value ≥1 and a fold change ≥1.5 & ≤ − 1.5 (B). Percentage depicts the proportion of transcripts from the transcriptomic analysis that are annotated in the Ingenuity canonical pathway. The percentage of transcripts that are downregulated or upregulated in each canonical pathway are depicted in green or red, respectively. (PDF 793 kb) [file 12890_2019_922_MOESM3_ESM.pdf]

**IgG control**

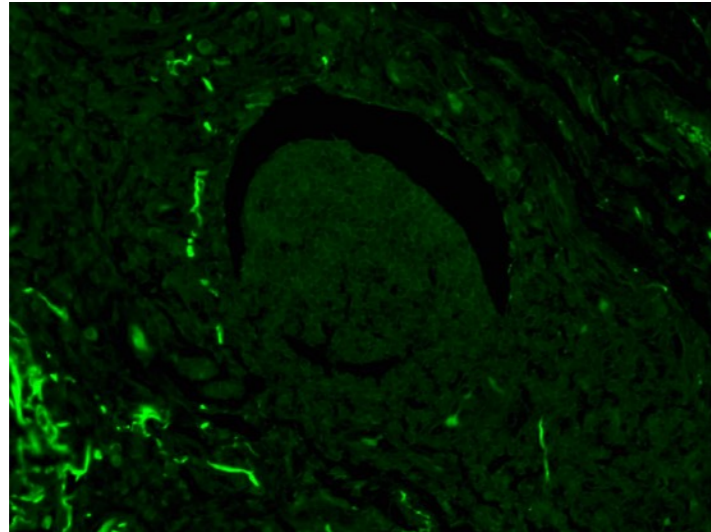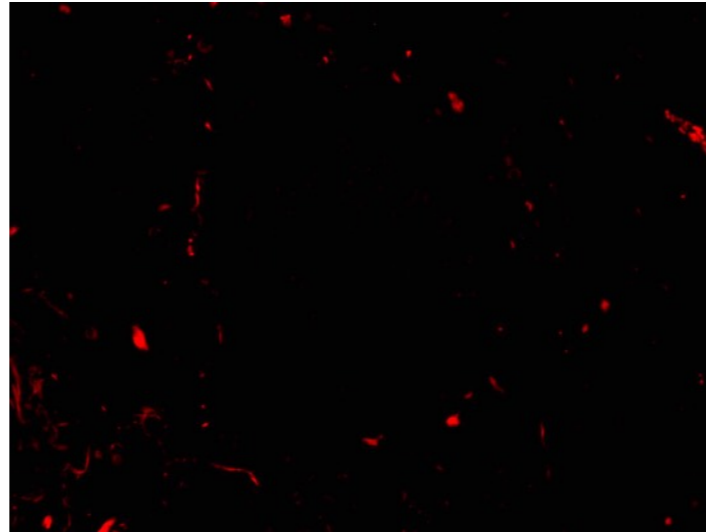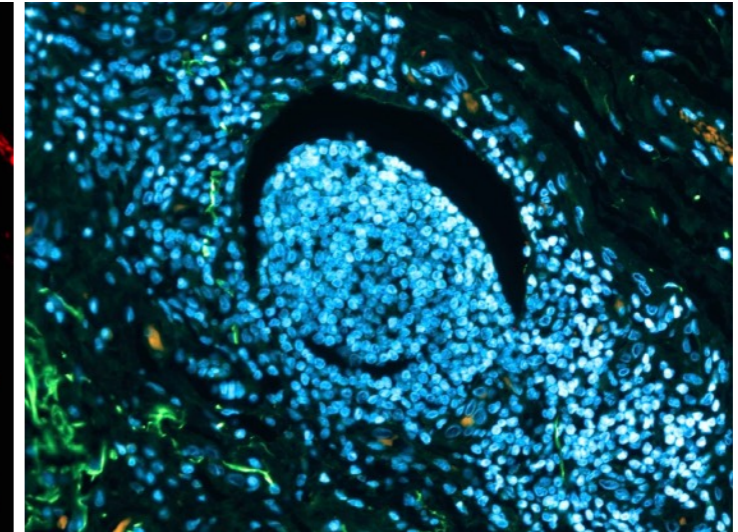

**Merged**

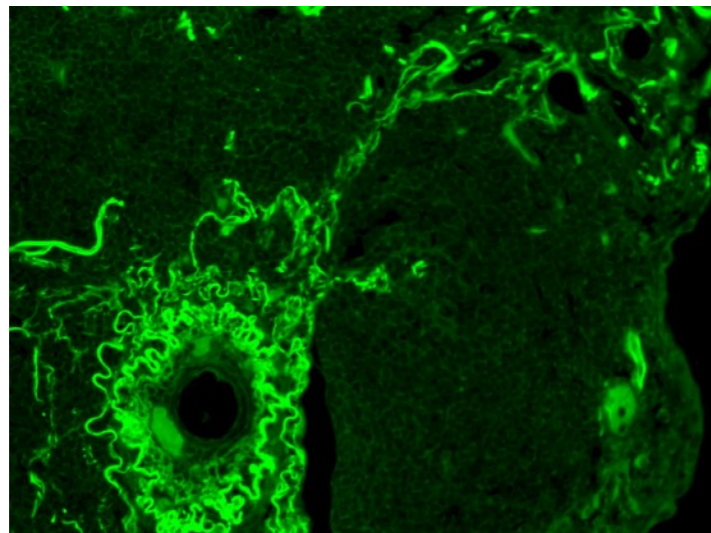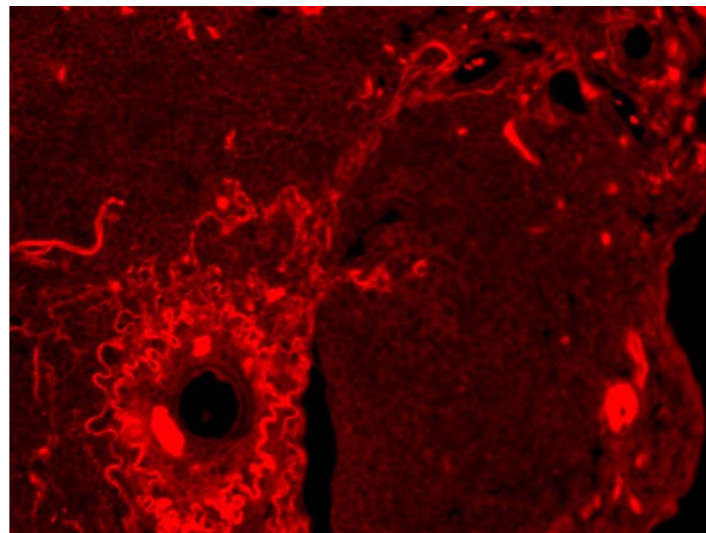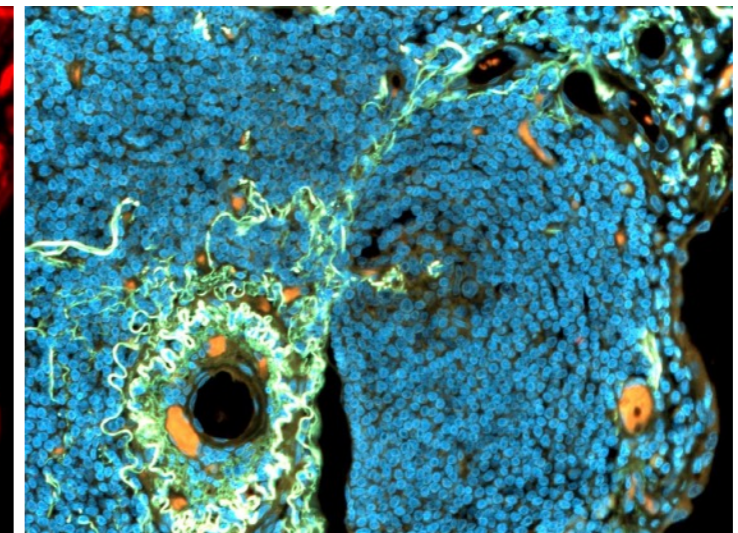

Supplement: Supplementary file 4 — Figure S4. Immunofluorescence IgG control staining of IPF lung tissues. Normal or IPF lung explants were stained IgG antibodies followed by fluorescently conjugated secondary antibodies and microscopy analysis. Representative images from two IPF patients are shown stained with IgG + Alexa Flour 488 conjugated secondary antibody (left), IgG + Alexa Flour 594 conjugated secondary antibody (middle) and the merged composite (right) acquired at 200x magnification. (PDF 405 kb) [file 12890_2019_922_MOESM4_ESM.pdf]
